# To the Editors of the Medical and Physical Journal

**Published:** 1805-11-01

**Authors:** 


### *To the Editors of the Medical and Physical Journal.*

GENTLEMEN,

### *To the Editors of the Medical and Physical Journal.*

GENTLEMEN,

UPON observing in your Journal for August, A Plan for the Extermination of the Small-pox, by Mr. Simmons, in the proposal of which, I doubt not, that gentleman is actuated by the purest motives of philanthropy, I take the liberty of remarking, that, as any compulsory method to effect so desirable an event may not be acceptable to the subjects of the British empire, it will be advisable to adopt some other mode of eradicating that dreadful evil. The following appears to me to be one of the most most eligible; and, as I do not know that it has been previously suggested, I humbly submit it, if deemed worthy of attention, to the consideration of your very numerous Readers.

As it is universally admitted, that the small-pox is highly contagious, and that the disorder which is introduced by inoculation is no less so than that which is taken in the natural way, no man is, I think, at liberty (considering the subject in a moral point of view) either to communicate the infection to his own children, till they are of a proper age to understand the nature of the disease, and consent to the operation, or to spread noxious effluvia in the neighbourhood, the evil consequences of which are too extensive to be calculated. But as, unhappily, moral considerations have not always their due weight with mankind, and as the prejudice in favour of variolous inoculation is still very great with some persons, I do not think it would be prudent entirely to prohibit the practice: I therefore propose, that every person who intends to inoculate with small-pox matter, shall be obliged by Government to take out a licence for that purpose, for which he shall pay the sum of one hundred pounds; and that whoever does introduce the small-pox by inoculation, without such licence, shall be subject to such pains and penalties as the Legislature, in its wisdom, shall think proper to appoint.

I am, &c.

*October* 2, 1805. CLERICUS.

---

